# Inhibitory potential of omega-3 fatty and fenugreek essential oil on key enzymes of carbohydrate-digestion and hypertension in diabetes rats

**DOI:** 10.1186/1476-511X-10-226

**Published:** 2011-12-05

**Authors:** Khaled Hamden, Henda Keskes, Sahla Belhaj, Kais Mnafgui, Abdelfattah feki, Noureddine Allouche

**Affiliations:** 1 Biotechnology High School of Sfax (ISBS), University of Sfax, Soukra Km 4.5; P.O. Box 261, Sfax 3038, Tunisia; 2 Laboratory of Animal Ecophysiology, University of Sfax, Faculty of Sciences of Sfax, P.O. Box 95, Sfax 3052, Tunisia; 3 Laboratory of Natural Products Chemistry, University of Sfax, Faculty of Sciences Of Sfax, 3000, P.B."1171", Sfax, Tunisia

## Abstract

**Background:**

diabetes is a serious health problem and a source of risk for numerous severe complications such as obesity and hypertension. Treatment of diabetes and its related diseases can be achieved by inhibiting key digestives enzymes-related to starch digestion secreted by pancreas.

**Methods:**

The formulation omega-3 with fenugreek terpenenes was administrated to surviving diabetic rats. The inhibitory effects of this oil on rat pancreas α-amylase and maltase and plasma angiotensin-converting enzyme (ACE) were determined.

**Results:**

the findings revealed that administration of formulation omega-3 with fenugreek terpenenes (Om3/terp) considerably inhibited key enzymes-related to diabetes such as α-amylase activity by 46 and 52% and maltase activity by 37 and 35% respectively in pancreas and plasma. Moreover, the findings revealed that this supplement helped protect the β-Cells of the rats from death and damage. Interestingly, the formulation Om3/terp modulated key enzyme related to hypertension such as ACE by 37% in plasma and kidney. Moreover administration of fenugreek essential oil to surviving diabetic rats improved starch and glucose oral tolerance additively. Furthermore, the Om3/terp also decreased significantly the glucose, triglyceride (TG) and total-cholesterol (TC) and LDL-cholesterol (LDL-C) rates in the plasma and liver of diabetic rats and increased the HDL-Cholesterol (HDL-Ch) level, which helped maintain the homeostasis of blood lipid.

**Conclusion:**

overall, the findings of the current study indicate that this formulation Om3/terp exhibit attractive properties and can, therefore, be considered for future application in the development of anti-diabetic, anti-hypertensive and hypolipidemic foods.

## Background

Diabetes mellitus is a major and growing public health problem throughout the world, with an estimated worldwide prevalence in 2008 more than of 347 million people and is a heterogeneous disorder with varying prevalence among different ethnic groups and it is reported to constitute the 16th leading cause of global mortality [1,2]. It is generally recognized that patients with diabetes are at risk for numerous severe complications, including diabetic obesity, hyperlipidemia and hypertension [3-5]. In this respect, many synthetic α-amylase and maltase inhibitors have been reported to reduce plasma glucose and lipid levels *via *delaying glucose absorption and retarding the liberation of glucose from oligosaccharides and disaccharides from dietary complex carbohydrates [6]. Nevertheless, the use of these inhibitors often induced disturbances in the gastrointestinal tract, including flatulence, diarrhea, and abdominal pain [6]. Accordingly, recent research seems to have granted special interest for the search of effective natural α-amylase and ACE inhibitors. In the same vein, essential oil isolated from plant sources, have attracted a great deal of attention in the biomedical arena particularly for their broad spectrum of therapeutic properties and relatively low toxicity [7,8]. Numerous studies have confirmed that consumption of omega-3 reduces the risk of developing chronic diseases such as diabetes and heart disease [9-11]. The benefits of omega-3 include anti-inflammatory and anti-oxidant effects[11]. Trigonella foenumgraecum has been shown to possess hypoglycaemic, anti-hypertensive and hypolipidemic activities in experimental animals as in human [9]. Fenugreek essential oil from the seeds of the *trigonella foenum gracecum *plant is rich in terpenenes. These nutrients, along with antioxidants, endow fenugreek essential oil with the power to fight viruses, cancer tumors, and free radicals which lead to aging. Recent studies have found that terpenenes can be absorbed through the gut and plays an important role in the control of cholesterol metabolism [12-14]. However, very little is known about cellular and biochemical mechanism of the anti-hyperglycemic and anti-hypertensive effect of fenugreek. Therefore, this study was undertaken to assess the effect of dietary fenugreek essential oil on key enzymes related to diabetes and hypertension, pancreas architecture and lipid profile in surviving diabetic rats.

## Materials and methods

### Extraction of terpenenes from fenugreek oil

The Fresh fenugreek seeds, purchased from a local market located at Sfax, Tunisia, were completely immersed in water and hydro-distilled for 4 hours in a Clevenger-type apparatus giving greenish-yellow oil. When the condensed material cooled down, the water and essential oils were separated. The oil was decanted to be used as essential oil. To improve its recovery, the essential oil was taken up in diethyl ether, dried over anhydrous sodium sulphate until the last traces of water were removed and stored in a dark glass bottle at 4°C until tested and analyzed. The extraction yield was 1.24% (w/w). To improve their quality, we add of this oil 15% of commercial omega 3 according of Pharmacy Central industry, Sfax, Tunisia).

### Gas chromatography-mass spectrometry (GC-MS)

The analysis of the fenugreek essential oil was performed on a GC-MS HP model 5975B inert MSD (Agilent Technologies, J&W Scientific Products, Palo Alto, CA, USA), equipped with an Agilent Technologies capillary DB-5MS column (30 m length; 0.25 mm i.d.; 0.25 mm film thickness), and coupled to a mass selective detector (MSD5975B, ionization voltage 70 eV; all Agilent, Santa Clara, CA). The carrier gas was He and was used at 1 mL min^-1 ^flow rate. The oven temperature program was as follows: 1 min at 100°C ramped from 100 to 260°C at 4°C min^-1 ^and 10 min at 260°C. The chromatograph was equipped with a split/splitless injector used in the split mode. The split ratio was 1:100. Identification of components was assigned by matching their mass spectra with Wiley and NIST library data, standards of the main components and comparing their Kovats Retention Indices (KRI) with reference libraries [15,16] and from the literature. The component concentration was obtained by semi-quantification by peak area integration from GC peaks and by applying the correction factors.

Total n-3 fatty acids were analyzed by gas chromatography as described previously [15,16] using a 28 component quantitative standard mixture (Prep 462; Nu-Chek Prep).

### Experimental induction of diabetes

Adult male Wistar rats, weighing 179 ± 10 g, and obtained from the Central Pharmacy, Tunisia, were employed in the study. The animals were kept in an environmentally controlled breeding room (temperature: 20 ± 2°C, humidity: 60 ± 5%, 12-hr dark/light cycle). All rats had free access to tap water and fasted overnight before blood and tissue collection. Diabetes was

induced in rats by a single intraperitoneal injection of freshly prepared alloxan solution in normal saline at a dose of 150 mg/kg body weight [5]. The rats were then kept for the next 24 hr on 5% glucose solution bottles in their cages to prevent hypoglycemia. After 2 weeks, rats with moderate diabetes having glycosuria and hyperglycemia (i.e., with blood glucose levels of 2 g/L) were chosen for the experiment. The handling of the animals was approved by the Tunisian Ethical Committee for the care and use of laboratory animals.

### Experimental procedure

A total of 60 rats (50 diabetic surviving rats and 10 control animals) were used. For diabetic rats, 1 month after alloxan injection and diabetes apparition, the day of beginning of experiments, 10 diabetic were sacrificed and referred as a diabetic rats before treatment (group 1) [(Diab(day0)] (glycemia 2 g/L). The other diabetic rats were divided into 5 groups: group 2, diabetic control rats named diabetic rats after treatment [Diab(day 60)]; group 3, diabetic rats treated with formulation: Omega-3 Fatty Acid Rich Fenugreek Essential Oil (5% in food) [Diab+For] [17]; group 4, diabetic rats treated with Fenugreek Essential Oil at a dose of 5% in food named [FO]; group 5, diabetic rats treated with omega 3 at a dose of 5% in food named [Om3] ]and group 6: normal rats were used as controls [Con]. Eight weeks after the beginning of oils administration to diabetic rats, the animals were sacrificed by decapitation, and the trunk blood collected. The serum was prepared by centrifugation (1,500 × g, 15 min, 4°C) the liver was removed, cleaned of fat; all these samples were stored at -80°C until used.

### Biochemical analysis

Alloxan, maltose, sucrose, and lactose were purchased from Sigma-Aldrich (St. Louis, MO, USA), the GOD, HDL, TC, TG and α-amylase kits were from Biomaghreb analyticals (Tunis, Tunisia). All other chemicals used were of analytical grade. The pancreas of each rat was excise. The pancreas was then homogenized and centrifuged (5,000 × g, 20 min). The supernatant was frozen and stored for further use in subsequent enzymatic assays. The activities of pancreas α-amylase and maltase activities were obtained by measuring the amount of glucose released from various substrates [18,19]. The plasma and kidney Serum ACE activity was measured using Hippuryl-His-Leu (HHL) as a synthetic substrate. For oral glucose and starch tolerance test, the carbohydrates loaded were as follows: glucose (2 g/kg) and starch (1 g/kg. These carbohydrates were orally administered via a gastric gavage route. Blood samples were collected from the tail vein at 0, 0.5, 1, and 2 h after the carbohydrate and oil administration. Total-cholesterol, triglyceride, albumin and HDL-cholesterol in the serum were measured using commercial kits from Biomagreb (Tunis, Tunisia). The level of total protein was determined by the method of Lowry *et al *using bovine serum albumin as the standard at 660 nm [20]. For histological studies, pieces of pancreas were fixed in a Bouin solution for 24 hours, and then embedded in paraffin. Sections of 5-μm thickness were stained with hematoxylin-eosin and examined under an Olympus CX41 light microscope.

### Statistical analysis

The data are presented as means ± SD. The determinations were performed from eight animals per group and the differences were examined by the one-way analysis of variance followed by the Fisher test (Stat View). Statistical significance was accepted at p < 0.05.

## Results

### Essential oil analysis

The chemical composition of fenugreek essential oil is presented in Table 1. The average yield in essential oil was 1.24% (w/w). GC-MS analysis resulted in the identification of 13 compounds representing 97.2% of the total essential oil. The most abundant components (> 4%) of the fenugreek essential oil were β-pinene (15.05%), 2,5-dimethylpyrazine (6.14%), 6-methyl-5-hepten-2-one (4.48%), camphor (16.32%), 3-octen-2-one (4.32%), β-caryophyllene (14.63%), neryl acetate (17.32%), α-selinene (4.04%) and geranial (4.81%). The identified compounds are known and were reported in a previous study (Table 1).

**Table 1:** Chemical composition of fenugreek essential oil.

**No**.	Compound	Composition (%)	Retention time (min)	KRI
1	β-Pinene	15.05	6.99	1023
2	2,5-Dimethylpyrazine	6.14	7.78	1103
3	6-Methyl-5-hepten-2-one	4.48	8.29	1206
4	α-Pinene	2.61	8.54	1109
5	γ-Terpinene	2.08	8.58	1251
6	Camphor	16.32	8.66	1514
7	3-Octen-2-one	4.32	8.87	1538
8	α-Campholenal	2.63	8.89	1471
9	β-Caryophyllene	14.63	8.92	1679
10	α-Terpineol	2.77	9.34	1677
11	Neryl acetate	17.32	9.56	1828
12	α-Selinene	4.04	9.89	1738
13	Geranial	4.81	9.99	1579
				
	Identification components (%)	97.2		
	Yield (%) (w/fw)	1.24		
				
	Omega 3 (18:3)	15% of total oil		

No: Numbers correspond to the peaks observed in the GC-MS chromatogram. KRI: Kovats Retention Index, retention index relative to n-alcane on DB-5MS Capillary column. w/fw: weight/fresh weight.

### Effect of the formulation Om3/terp on α-amylase activity in plasma and pancreas and blood glucose level of control and diabetic rats

**Figure 1:**
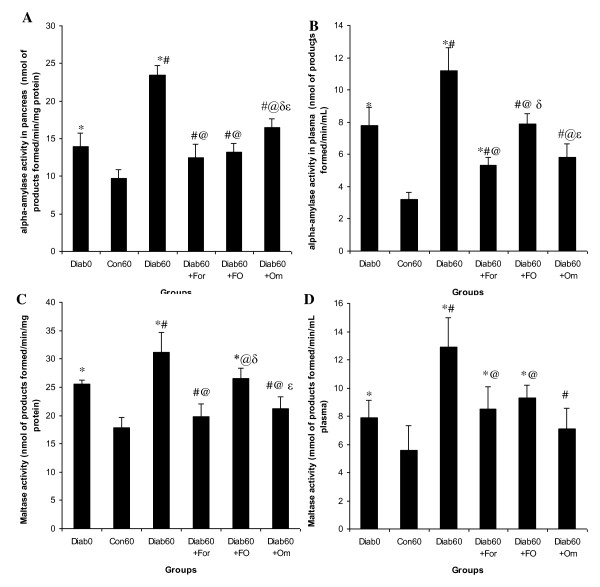
**Inhibitory effect of formulation Om3/terp on α-amylase and maltase activities in pancreas and plasma and blood glucose rate on surviving diabetic rats**. Values are given as mean ± SD for group of 10 animals each. Values a re statistically presented as follows: *P < 0.05 significant differences compared to controls. ^#^P < 0.05 significant differences compared to diabetic rats day0. ^@^P < 0.05 significant differences compared to diabetic rats day60. δp < 0.05 significant differences compared to diabetic rats treated with omega-3 fatty acid rich omega-3 fatty acid rich fenugreek essential oil. ^ε^P < 0.05 significant differences compared to diabetic rats treated with FO.

**Figure 2:**
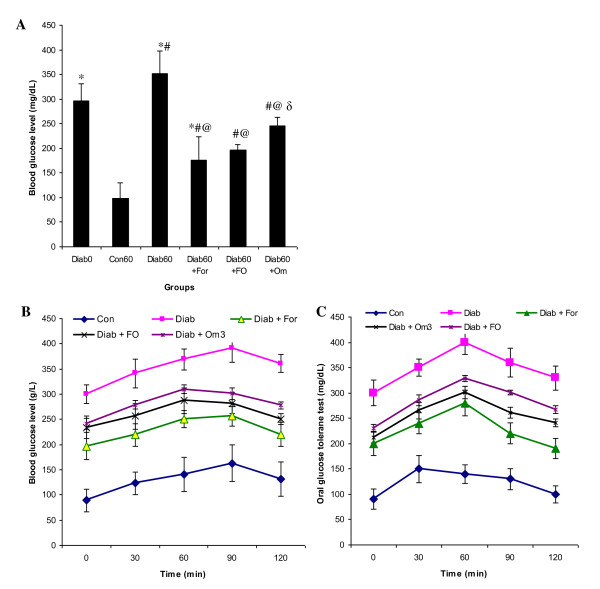
**Effect of formulation Om3/terp on glucose (A) and oral starch tolerance test (B) in of control and experimental groups of rats**. Statistical analyses as given in Figure legend 1.

The findings indicated that compared to the control, there was a significant increase in the activities of α-amylase and maltase in pancreas and plasma of diabetic rats, which leads to significant increase in plasma glucose rate by 259%. However, the administration of the formulation Om3/terp to surviving diabetic rats is associated by considerable reductions in the plasma and pancreas α-amylase and maltase activities. This potential inhibitory effect of the formulation Om3/terp on α-amylase and maltase activities in both plasma and pancreas was confirmed by a reduction of blood glucose rate by 51% (Figure 1 &2).

### Effect of the formulation Om3/terp on oral carbohydrate tolerance test (OCTT) in diabetic rats

With the intent to assess the effect of orally administered the formulation Om3/terp on systemic glucose homeostasis and confirmed the potential inhibitory action of key digestive enzymes on carbohydrate digestion secreted by pancreas, we performed an oral glucose and Starch tolerance test in conscious fasted rats after the formulation Om3/terp administration. These results clearly showed that, acute oral administration of the formulation Om3/terp reduced significantly peak glucose concentration 60 min after glucose and starch administration as compared to untreated diabetic rats (Figure 2).

### Effect of the formulation Om3/terp on plasma and liver total n-3 fatty acids concentration

**Figure 3:**
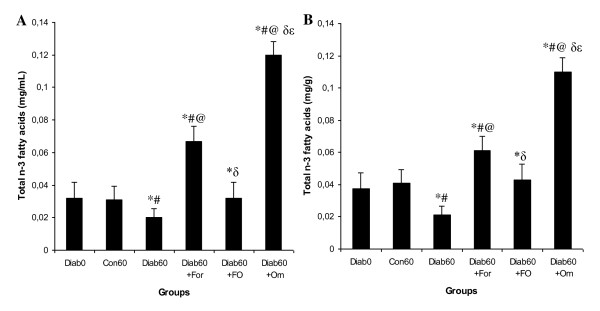
**Change in the plasma and liver omega fatty acids in diabetic rats treated with formulation Om3/terp**. Statistical analyses as given in Figure legend 1.

Figure 3 indicates that, when compared to the non-diabetic rats, the total n-3 fatty acids concentrations in plasma and liver decreased significantly in the diabetic rats. Interestingly, in diabetic rats, the administration of the formulation Om3/terp greatly increased the total n-3 fatty acids levels in both plasma and liver (Figure 3).

### Effect of the formulation Om3/terp on plasma and liver lipid concentration

Figure 5 indicates that hyperglycaemia associated by remarkable increases in the total-cholesterol, triglyceride and LDL-cholesterol concentrations in the plasma and liver and decrease in the HDL-cholesterol compared to that of the non-diabetic rats (Table 2). However, the administration of the formulation Om3/terp to surviving diabetic rats was reverted back the rate of the total-cholesterol, triglycerides and LDL-cholesterol in the plasma and liver. Moreover, fenugreek omega-3 fatty acid rich fenugreek essential oil supplement to surviving diabetic rats increases the HDL-cholesterol rate in plasma and liver of diabetic rats.

**Table 2:** Total cholesterol (TC), LDL-cholesterol (LDL-C), HDLcholesterol (HDL-C) and triglycerides (TG) in serum and liver of diabetic rats treated with formulation Om3/terp.

Groups	T-C	HDL-C	LDL-C	TG
***Serum (g/L)***				
Control	1.42 ± 0.12	0.63 ± 0.07	0.97 ± 0.05	0.57 ± 0.06
Diab 0day	1.87 ± 0.31*	0.57 ± 0.06*	1.30 ± 0.08*	0.98 ± 0.10*
Diab 60 day	2.61 ± 0.41^*#^	0.41 ± 0.02^*#^	2.20 ± 0.17^*#^	1.54 ± 0.16^*#^
Diab + For60	1.59 ± 0.21*^@^	0.78 ± 0.08*^#@^	1.13 ± 0.11*^#@^	0.74 ± 0.05*^#@^
Control+ FO	2.18 ± 0.22*^@δ^	0.54 ± 0.04*^@δ^	1.76 ± 0.06*^#δ^	0.61 ± 0.07^#@δ^
Diab + Om60	1.86 ± 0.22*^@δε^	0.83 ± 0.04*^#@ε^	1.36 ± 0.06*^#δ^	0.61 ± 0.07^#@δ^
***Liver (mg/g)***				
Control	1.1 ± 0.12	0.48 ± 0.03	0.62 ± 0.06	0.41 ± 0.06
Diab 0day	1.94 ± 0.21*	0.42 ± 0.03*	1.52 ± 0.17*	0.49 ± 0.05*
Diab 60 day	2.62 ± 0.30^*#^	0.33 ± 0.05^*#^	0.63 ± 0.07	0.89 ± 0.08^*#^
Diab + For60	1.72 ± 0.31^*#@^	0.61 ± 0.05*^#@^	2.29 ± 0.29*^#@^	0.51 ± 0.06^@^
Control+ FO	1.64 ± 0.10^#@δ^	0.44 ± 0.07^@δ^	0.30 ± 0.04*^#@δ^	0.47 ± 0.05^@^
Diab + Om60	1.24 ± 0.10^#@δ^	0.65 ± 0.07*^#@ε^	0.54 ± 0.04^#@δε^	0.36 ± 0.05^#@δ^

### Effect of the formulation Om3/terp on pancreas architecture

**Figure 4:**
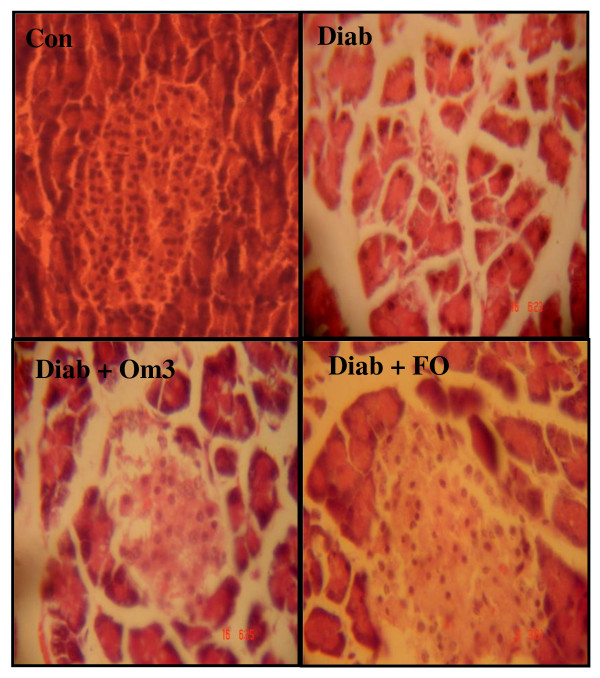
**Effect of formulation Om3/terp administration to surviving diabetic rats on pancreas architecture**. Figure 3 presents the histopathological examination of pancreas. In control rat, the pancreas shows normal islets (Con). In alloxan-treated rats pancreas a severe β-Cells atrophy was shown where the most pancreatic islets were completely empty after 8 weeks of alloxan administration (Diab day60). In formulation Om3/terp treated diabetic rats; a patent protective action of β-Cells was shown and only initial stages of atrophy of β-Cells were observed (Diab + For).

The data indicated that compared to the case of the control rats, alloxan exhibited β-Cells degeneration in the pancreas of diabetic. A potent protective action of β-Cells was, however, recorded in the diabetic rats treated with the formulation Om3/terp (Figure 4).

### Effect omega-3 fatty acid rich fenugreek essential oil on plasma ACE activity

**Figure 5:**
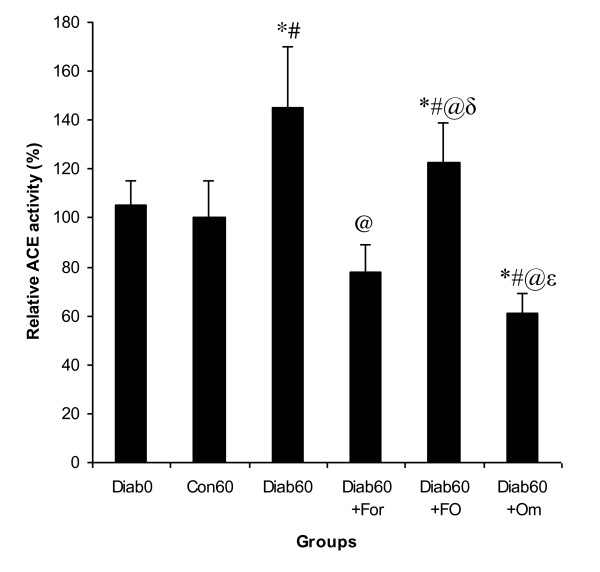
**Relative activity of ACE in plasma of control and experimental groups of rats**. Statistical analyses as given in Figure legend 1.

Figure 5 indicates that the plasma ACE activity in the diabetic rats underwent a potent increase of 45% as compared to the non-diabetic rats. However, the administration of the formulation Om3/terp to surviving diabetic rats was reverted back the activity of ACE in plasma back by 38%.

## Discussion

Dietary carbohydrate in human is a major nutrient and α-amylase play an important role in carbohydrate digestion. It is generally recognized that carbohydrates are digested into oligosaccharides by α-amylase and then into maltose by enzymes secreted by the digestive tract. Maltose is then converted into glucose by maltase located in the small intestinal mucosa [21-23]. It was reported that many α-amylase inhibitors may reduce postprandial plasma glucose level via retarding the liberation of D-glucose of oligosaccharides and disaccharides from dietary complex carbohydrates and delaying glucose absorption. However, usefulness of these α-amylase inhibitors often induced disturbances of the gastrointestinal tract, including flatulence, diarrhea, and abdominal pain [24].

Many studies have shown effects of different dietary constituents on α-amylase and maltase activity during diabetes. Dietary spices or their active principles have been shown to have a positive influence on activities of disaccharidases. Our studies have shown a significant improvement in pancreas and plasma α-amylase and maltase activity in fenugreek essential oil-fed diabetic groups when compared to untreated-diabetic groups. In this study, a highest level obtained in the fenugreek essential oil is Neryl acetate (17%), Camphor (16%) and β-Pinene (15%). The terpenes such as β-pinene which exist in fenugreek essential oil inhibited key enzymes related to type 2 diabetes principally α-amylase and maltase in both pancreas and plasma and consequently hyperglycemia (Figure 1, 2). In fact, recent research has reported that administration of terpenenes to diabetic exert blood glucose lowering effect and high antioxidant activity in alloxan-induced diabetic rat [12-15]. Therefore, minimally processed and phytochemical enriched plants such as vegetables, fruits, nuts, seeds, and grains generally increase postprandial glucose and triglycerides to a lesser degree than do processed foods [12-15]. Moreover, this study showed that administration of fenugreek essential oil to surviving diabetic rats restores the structure of pancreas βcells resulting in the increase of insulin secretion which decreases glucose level in plasma. Both the inhibitory effect of key enzymes related to starch digestion and absorption of starch and the regeneration effect of pancreas β-cells by fenugreek essential oil prevent the increase of blood level as antidiabetic action. Others studies have reported that administration of herbal terpenes such as β-pinene, existed in fenugreek oil (Table 1) to surviving diabetic rats protect the architecture of pancreas β-cells, preserve the insulin secretion and stimulate the regeneration of this type of cells [22,23].

Additionally, this study showed that administration of formulation Om3/terp to surviving diabetic rats was associated by lower level of TG and total-cholesterol and higher rate of HDL-cholesterol in plasma of diabetic rats [22,23]. Diabetic rats treated with omega-3 fatty acid rich fenugreek essential oil show a therapeutic action. In fact, a significant decrease in TCh and TG content and increase in HDL-Ch rate were observed in both plasma and hepatic tissues. This hypolipidemic and hypercholesterolemia effect of formulation Om3/terp probably resulted to omega 3, which exert potential hypolipidemic activity by their capacity[24] to i) inhibition of key enzyme related to cholesterol synthesis and transfer such as 3-Hydroxy-3-methylglutaryl (HMG)-CoA reductase and acyl-CoA:cholesterol acyltransferase (ACAT) activities [25] ii) theirs role in the control of peroxisome proliferator-activated receptor α (PPAR-α) that controls the expression of genes involved in hepatic fatty acid oxidation and the transcription factor SREBP1c that is required for suppression of de novo lipogenesis and monounsaturated fatty acids synthesis [26] iii) inhibition of intestinal lipase activity, which leads to decrease of lipid digestion and absorption in intestine as hypolipidemic action [4].

The ACE plays a dominant role in the regulation of the water electrolyte balance and blood pressure. Activation of this system has been considered to be a main cause of renovascular hypertension [27,28]. The physiological function of ACE is related to the regulation of blood pressure and electrolyte homeostasis by converting angiotensin I (Ang I) into potent vasoconstrictor angiotensin II and by inactivating bradykinin The importance of ACE inhibitors in the chronic treatment of various cardiovascular diseases such as hypertension, congestive heart failure, myocardial infarction, diabetic nephropathy, or renal dysfunction is now well established. In fact, inhibitors of ACE such as Captopril, Enalapril, Lisinopril and Temocapril are widely used in the clinic for the treatment of hypertension. Moreover, variations of serum ACE activity have been reported in pathologies involving either a stimulation of monocyte cell line or an endothelial abnormality [28]. This study showed that diabetes was associated with increase of plasma ACE activity and this activity was decreased significantly by addition of formulation Om3/terp and this action probably resulted to omega-3 fatty acids existing in the fenugreek essential oil [12]. The important findings in this study were that fenugreek essential oil has an inhibitory effect on ACE. These result in accord with the results of Balaraman *et al *[28] and Das [29,30], where showed that administration of omega-3 fatty to experimentally induced hypertension in rat reduced blood pressure in fructose-induced hypertensive rats. Thus, fenugreek seeds exhibit a significant antihypertensive effect. The mechanism of action may partly involve the serotonergic antagonistic property involving the 5-HT2 receptor subtype.

## Competing interests

The authors declare that they have no competing interests.

## Authors' contributions

KH: design, coordination of fenugreek oil extraction, rats treatment and biochemical and histological analysis. HK: extraction and chemical composition of fenugreek essential oil. SH: participated in animal treatment. KM: participated of omega-3 fenugreek essential oil formulation. AF: participated in its design and coordination. NA: participated in its design and coordination. All authors read and approved the final manuscript.
